# Dominant Plant Functional Group Determine the Response of the Temporal Stability of Plant Community Biomass to 9-Year Warming on the Qinghai–Tibetan Plateau

**DOI:** 10.3389/fpls.2021.704138

**Published:** 2021-09-03

**Authors:** Chengyang Li, Chimin Lai, Fei Peng, Xian Xue, Quangang You, Feiyao Liu, Pinglin Guo, Jie Liao, Tao Wang

**Affiliations:** ^1^Key Laboratory of Desert and Desertification, Northwest Institute of Eco-Environment and Resources, Chinese Academy of Sciences (CAS), Lanzhou, China; ^2^College of Resources and Environment, University of Chinese Academy of Sciences, Beijing, China; ^3^Arid Land Research Center, Tottori University, Tottori, Japan; ^4^Beiluhe Observation and Research Station of Frozen Soil Engineering and Environment, State Key Laboratory of Frozen Soil Engineering, Northwest Institute of Eco-Environment and Resources, Chinese Academy of Sciences (CAS), Lanzhou, China; ^5^Drylands Salinization Research Station, Key Laboratory of Desert and Desertification, Northwest Institute of Eco-Environment and Resources, Chinese Academy of Sciences (CAS), Lanzhou, China

**Keywords:** climate change, alpine meadow, aboveground biomass, Temporal stability of biomass, soil water availability

## Abstract

Ecosystem stability characterizes ecosystem responses to natural and anthropogenic disturbance and affects the feedback between ecosystem and climate. A 9-year warming experiment (2010–2018) was conducted to examine how climatic warming and its interaction with the soil moisture condition impact the temporal stability of plant community aboveground biomass (AGB) of an alpine meadow in the central Qinghai-Tibetan Plateau (QTP). Under a warming environment, the AGB percentage of grasses and forbs significantly increased but that of sedges decreased regardless of the soil water availability in the experimental plots. The warming effects on plant AGB varied with annual precipitation. In the dry condition, the AGB showed no significant change under warming in the normal and relatively wet years, but it significantly decreased in relatively drought years (16% in 2013 and 12% in 2015). In the wet condition, the AGB showed no significant change under warming in the normal and relatively drought years, while it significantly increased in relatively wet years (12% in 2018). Warming significantly decreased the temporal stability of AGB of plant community and sedges. Species richness remained stable even under the warming treatment in both the dry and wet conditions. The temporal stability of AGB of sedges (dominant plant functional group) explained 66.69% variance of the temporal stability of plant community AGB. Our findings highlight that the temporal stability of plant community AGB is largely regulated by the dominant plant functional group of alpine meadow that has a relatively low species diversity.

## Introduction

The global surface temperature has remarkably increased by 0.85°C from 1880 to 2012. It is predicted that the temperature will continue to rise 0.3–4.8°C by the end of the 21st century (IPCC, 2013), especially in high altitudes and latitude regions (Chen et al., 2013). The Qinghai-Tibetan Plateau (QTP), with an average elevation >3,000m, is the highest and largest plateau on the earth (Li et al., 2018). The annual mean temperature on the QTP will increase by 2.8–4.9°C at the end of the 21st century (IPCC, 2013). Besides, extreme drought/wet events are also increasing regardless of the no change in the total precipitation (Chen et al., 2015).

In general, climate warming can alleviate the temperature constraint on the plant growth, thus result in an increase of plant productivity and change the plant community composition in cold regions (Elmendorf et al., 2012). As the plant growth is regulated by the combination of temperature and available water, various even divergent responses of plant productivity to the climate warming were reported in the tundra, mountainous alpine ecosystems with different water availability (Walker et al., 2006; Wu et al., 2011; Elmendorf et al., 2012; Liu et al., 2018). The warming can induce a higher ecosystem evapotranspiration (Ganjurjav et al., 2016), therefore depletes soil moisture. In a cold and wet environment, the warming-induced change in soil moisture is not enough to limit the plant growth, thus sustain a consistent enhancement of plant productivity, while in a cold and dry environment plant growth may be suppressed by the reduction in soil moisture resulted from the warming (Li et al., 2018; Liu et al., 2018). For example, warming stimulates community biomass by increasing shrubs and graminoids biomass in tundra (Elmendorf et al., 2012) and plant height in an alpine meadow (Ganjurjav et al., 2016). By contrast, warming-induced water stress inhibits community biomass by reducing the cover of graminoids and forbs in an alpine steppe (Ganjurjav et al., 2016) and grasses biomass in a semi-arid grassland (Yang et al., 2011).

The response of different plants to warming determines the community level productivity in a warmer climate. Warming-induced change in soil moisture condition can mediate plant community composition, which may be partly attributable to the various root characteristics of different plant functional groups (Xu et al., 2020). Deep root species can use soil moisture in upper and deeper layers and have higher drought tolerance (Xu et al., 2018). A 4-year warming and drought experiment in a mesic alpine grassland on the northeastern QTP found that plant community composition gradually shifts from more sedges species to more graminoids and forbs species under warming and drought treatments (Liu et al., 2018). Graminoids and forbs species generally have deeper roots than sedges species (Li et al., 2018, 2021; Xu et al., 2018), making them better deal with the environment with limited soil water availability (Klein et al., 2007). These findings suggest that the response of different plant functional groups to warming may depend on soil water availability and functional traits. Therefore, the combined effect of temperature and water availability needs to be considered when exploring plant biomass and community composition responses to warming in different ecosystems. Meta-analyses studies suggest that the responses of plant biomass and community composition to warming vary with time and site (Elmendorf et al., 2012; Liu et al., 2018). For instance, the aboveground biomass (AGB) was increased in the first year of warming, while decreased after 5years of warming in an alpine meadow on the QTP (Li et al., 2004), which suggest that warming may increase the interannual variability of AGB and decrease the temporal stability of plant community AGB (Quan et al., 2021). Without the consideration of the ecosystem functional stability, the accuracy of terrestrial ecosystem models in predicting future ecosystem service and terrestrial feedback to climate will be hampered (Oehri et al., 2017; Sasaki et al., 2019). The response of the productivity of alpine meadow, one of the major biomes on the QTP, to climate change, therefore, is crucial for understanding the natural processes and for the sustainability of pastoral agriculture on the QTP (Harrison et al., 2014; Zhou et al., 2019).

The stability of productivity may be affected by several mechanisms. Firstly, ecosystem functions and services are more stable with higher diversity due to both the “sampling effect” and “compensatory effect” in functionally similar groups (Tilman et al., 2014; Pennekamp et al., 2018), and the insurance theory in functionally dissimilar groups (Tilman, 1996; Zavaleta et al., 2010; Isbell et al., 2011). The “compensatory effect” or the “niche complementarity effect” increases the total resource use efficiency by spatial and temporal partitioning of resource use (Jiang and Pu, 2009). In the “sampling effect,” the higher likelihood of the presence of productive species in the diverse community could enhance the resistance of community to environmental variations, therefore, maintain the high stability (Zhang and Zhang, 2006). Secondly, the mass-ratio hypothesis indicates that the temporal stability of plant community biomass is mainly controlled by dominant species (Grime, 1998; Ma et al., 2017). The dominant species usually have high canopy height and large specific leaf area (Mariotte, 2014), and are fewer in population, more expansive in morphology, and form a large proportion of the biomass (Yang et al., 2017). In contrast, subordinates and rare species are usually more numerous in population than the dominants, smaller in stature, and account for a lower proportion of the biomass (Yang et al., 2017). Therefore, changes in the temporal stability of dominant species may greatly affect the temporal stability of plant community AGB. Thirdly, the temporal stability of plant community AGB could vary with environmental context. For example, the strong environmental filtering selection for species that adapt to drought (e.g., C_4_ species) may lead to the decrease of the variability of plant community, which increased the temporal stability of plant community AGB in the dry condition compared to wet condition (García-Palacios et al., 2018). In recent decades, the response of these stabilizing factors to climate change has been paid growing attention, and changes in these stabilizing factors consequently affect the temporal stability of plant community AGB (Shi et al., 2016).

Alpine meadow is dominated by shallow-rooted sedges on the QTP (Ganjurjav et al., 2016). Climate warming could decrease the sedges abundance because warming aggravates water stress in the top soil layer (Ganjurjav et al., 2016), which might reduce the temporal stability of plant community AGB (Quan et al., 2021). But previous studies have reported inconsistent effects of warming on the temporal stability of plant community AGB in alpine meadow on the QTP. For example, in a site with mean annual precipitation of 747mm, Quan et al. (2021) reported that warming reduces the temporal stability of plant community AGB by decreasing dominant species’ stability and co-existing species compensatory dynamics. However, in an area with annual precipitation ranges from 280 to 530mm, Zhou et al. (2019) found that warming had little effect on the temporal stability of plant community AGB, primarily because the negative effect of daytime warming on plant community stability can be offset by the positive effect of night warming. Therefore, it is far from clear how the temporal stability of plant community biomass responds to warming and the underlying mechanisms and whether the warming effects on the temporal stability of plant community AGB vary with soil water availability. A better understanding of such knowledge is crucial for the sustainable development of alpine ecosystem.

In this study, we conducted a 9-year warming experiment with different soil moisture conditions in the permafrost region of the QTP. Specifically, the objectives of this study were (1) to explore warming effects on the plant AGB, community composition and the temporal stability of plant community AGB in the dry and wet conditions; and (2) to determine the mechanism that control the response of the temporal stability of plant community AGB to warming in the different water conditions.

## Materials and Methods

### Study Site

**Figure 1:**
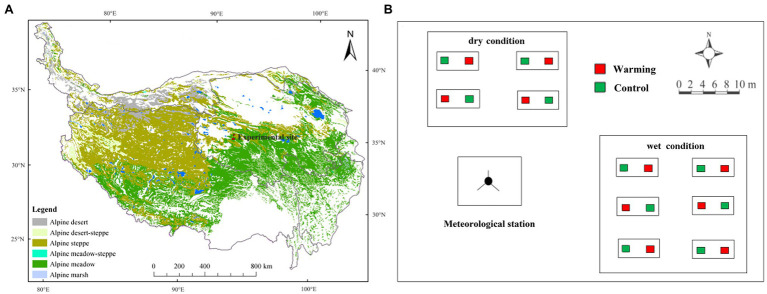
The location of the experimental site **(A)**, and layout of experimental design **(B)**.

A 9-year field warming manipulation experiment was carried out to investigate the temporal stability of vegetation productivity to climatic warming in both relatively dry and wet conditions. This study was conducted at the Beiluhe Permafrost Observation station, Chinese Academy of Science (92^o^56E', 34^o^49'N; Figure 1A). The station is in the Yangtze River’s source region on the QTP, with a mean elevation of 4,635m. The mean annual temperature and mean potential annual evaporation is −3.8°C and 1316.9mm, respectively. The experimental site is a permafrost region with an active layer thickness of 2–3.2m, and the permafrost period lasts from September to April (Wang et al., 2007). Plant roots in the 0–20cm soil layers accounted for more than 80% of the total root biomass (0–50cm layer; Li et al., 2018). The study site is dominated by alpine meadow species, such as *Kobresia pygmaea* (Sedge), *Kobresia capillifolia* (Sedge), and *Carex moorcroftii* (Sedge). Some forbs species like *Polygonum viviparum* are also widely distributed. The mean plant height at a community level is 5–10cm. The experimental field was on a mountain slope with a mean inclination of 5°.

### Experimental Design

The warming experiment began in 2010. In this study, we conducted a completely randomized split-plot experimental design (Figure 1B). In the design, soil moisture condition was used as the splitting factor and warming was the main treatment. More detailed information about soil properties (0–20cm depth) and plant features for the sampling plots in the different soil moisture conditions were shown in Supplementary Table S1. The distance between the dry and wet condition varied from 20 to 50m. The average elevation in the dry condition was 1m higher than in the wet condition. The annual mean soil moisture at the lower slope location (wet condition; 12.09%, v/v%) was significantly higher than the upper slope location (dry condition; 6.76%, v/v%) at the top layer (0–10cm) over 2010–2018.

There are four control plots and four warmed plots in the dry area, and six control plots and six warmed plots in the wet area. In total, 20 treatment plots were established. In the middle of each warming plot, one 165cm×15cm infrared heater (MR-2420, Kalglo Electronics Inc., Utah, United States) with an output of 150W m^−2^ was installed. A “dummy” radiator (no heating element) in each control plot was installed to eliminate any effects of shading by heaters in control plots (Li et al., 2018). Experimental warming increased daily mean soil temperature (at 10cm depth) by 1.8°C compared to control plots (Xue et al., 2014). All the warming plots were heated yearly-round since July 1st, 2010. From July 2013, there was a malfunction of the substation and no power was available until May 2014. The precipitation and air temperature (2m) from the ground were automatically recorded by a micro-meteorological observation station.

### Soil Temperature and Moisture Measurements

Soil temperature was measured by a thermo-probe (Model 109, Campbell Scientific, Inc., Utah, United States), which was installed at the top layer (0–10cm) and the deep layer (30–40cm) in the center of each plot. A frequency domain reflectometry (FDR; EnviroSmart sensor, Sentek Pty Ltd., Stepney, Australia) was installed at depths of 10 and 40cm to monitor the soil water content (v/v%). A CR-1000 data logger (Campbell Scientific, Inc., Utah, United States) was used to record the soil temperature and moisture every 10-min interval. The daily average soil temperature and moisture were averaged by the 10-min recorded data. The daily recorded data were then averaged into mean annual data and mean August data. Because no power was available from July 2013 until May 2014, soil temperature and moisture were recorded from July 2010 until June 2013 and June 2014 until December 2018.

### Vegetation Characteristic Measurements

In late August of every year (from 2010 until 2018), we used the following methods to determine each plot’s vegetation characteristics. Each plot (2m×2m) was divided equally into four parts diagonally. The height of each functional group (grasses, sedges, and forbs) was randomly measured 10 times in each part, respectively. A frame with interior dimensions of 27cm×27cm was used to measure the coverage of each functional group in each part. The height and coverage of each plot were obtained by averaging the heights and coverage from the four parts. Besides, in July and September of 2017, and June of 2018, 20 quadrats were investigated every month. In each plot, the aboveground part of each functional group was clipped (27cm×27cm), and then dried in an oven to obtain the AGB. Then the dried AGB of each functional group was fitted against the average height and coverage using a multiple linear regression. The AGB of each functional group in each August (from 2010 until 2018) was estimated using the multiple regressions (Eqs. 1–3). The community AGB was obtained by the sum of AGB of sedges, forbs, and grasses.

(1) $$\begin{array}{c} \text{AGB}\;\text{of}\;\text{sedges}=157.24c-8.13h+59.56\;\\ \left(R^{2}=0.362;;p<0.001;;n=60\right) \end{array}$$

(2) $$\begin{array}{c} \text{AGB}\;\text{of forbs}=158.60c-13.52h+35.57\;\\ \left(R^{2}=0.405;;\;p<0.001;;\;n=60\right) \end{array}$$

(3) $$\begin{array}{c} \text{AGB}\;\text{of grasses}=91.63c-0.72h+17.72\;\\ \left(R^{2}=0.332;;\;p<0.001;;\;n=60\right) \end{array}$$

Where, *c* is the coverage (%); *h* is the height (cm); and *n* is the number of plots used for establishing this multiple regression.

According to each species’ ecological niches or functions, all species were classified into grasses, sedges, and forbs. The three functional groups differ in rooting depths. Grasses and forbs species generally have deeper roots that are more than 30cm, while sedges have shallower roots that are less than 20cm (Xu et al., 2018; Li et al., 2021).

### Species Richness and Temporal Stability of AGB

We calculated the species richness (species richness; Sasaki and Lauenroth, 2011; Ma et al., 2017) from 2010 to 2018 to reflect the diversity. The species richness is the total number of plant species in each plot.

The temporal stability of the community and each functional group’s AGB were calculated as the reciprocal coefficient of variation (ICV) of biomass (Tilman, 1996; Xu et al., 2015). Greater ICV means higher stability (Zhang and Zhang, 2006).

(4) Temporal stability=ICV=Mean/SD

Where, ICV is the reciprocal coefficient of variation of community and each functional group’s AGB; SD is SD of community and each functional group’s AGB; Mean is mean biomass of community and each functional group’s AGB.

### Data Analysis

Repeated-measures ANOVA was used for testing the effects of warming, soil moisture condition, year, and their interaction on community AGB, the AGB of grasses, sedges, and forbs. The same analysis was used for testing the effects of warming, soil moisture condition, year, depth, and their interaction on soil temperature and soil moisture. The community AGB, AGB of grasses, sedges, and forbs were log_10_-transformed. The Tukey’s honestly significant difference (HSD) test was conducted to examine the significant effect of warming on soil temperature, soil moisture, community AGB, the AGB of grasses, sedges, and forbs in different years under dry and wet conditions. Two-way ANOVA was used to explore the effects of warming, soil moisture condition and their interaction on the temporal stability of the community AGB, AGB of sedges, forbs, and grasses. The correlation between the temporal stability of plant community AGB and the temporal stability of AGB of grasses, sedges, forbs, and species richness were examined by simple linear regression analyses. The combined effects of species richness, the temporal stability of AGB of grasses, sedges, and forbs on the temporal stability of plant community AGB were evaluated by general linear models (GLMs).

## Results

### Precipitation and Temperature During the Experiment Period

**Figure 2:**
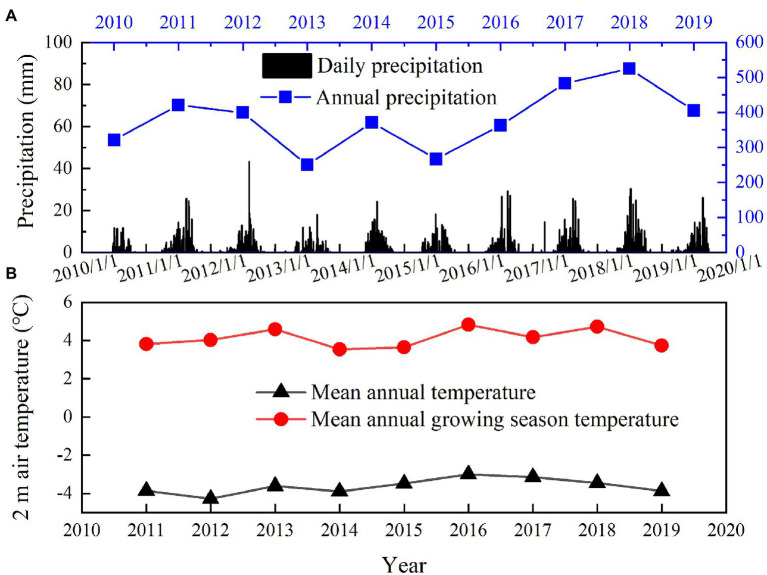
Variations in mean daily precipitation and annual precipitation from 2010 to 2019 **(A)**, mean annual temperature and mean growing season temperature (May–September) from 2011 to 2019 **(B)**.

Based on meteorological station data, over 95% of the annual precipitation falls from May to October (Figure 2A). The annual precipitation ranges from 250 to 525mm during the experimental period. The lowest annual precipitation was observed in 2013 (250mm) and 2015 (267mm; Figure 2A), which was 35 and 30% lower than the mean annual precipitation (382mm, 2002–2019) in this area. The highest annual precipitation was observed in 2017 (483mm) and 2018 (525mm; Figure 2A), which was 27 and 37% higher than the mean annual precipitation. Therefore, 2013 and 2015 were considered as relatively drought years, and 2017 and 2018 were considered as relatively wet years. The mean annual temperature is −3.62°C and mean growing season temperature (May–September) is 4.12°C (2011–2019; Figure 2B). The mean annual temperature ranges from −4.28 to −3.00°C and mean growing season temperature ranges from 3.55 to 4.83°C during the experimental period (2011–2019; Figure 2B).

### Soil Microclimate

**Figure 3:**
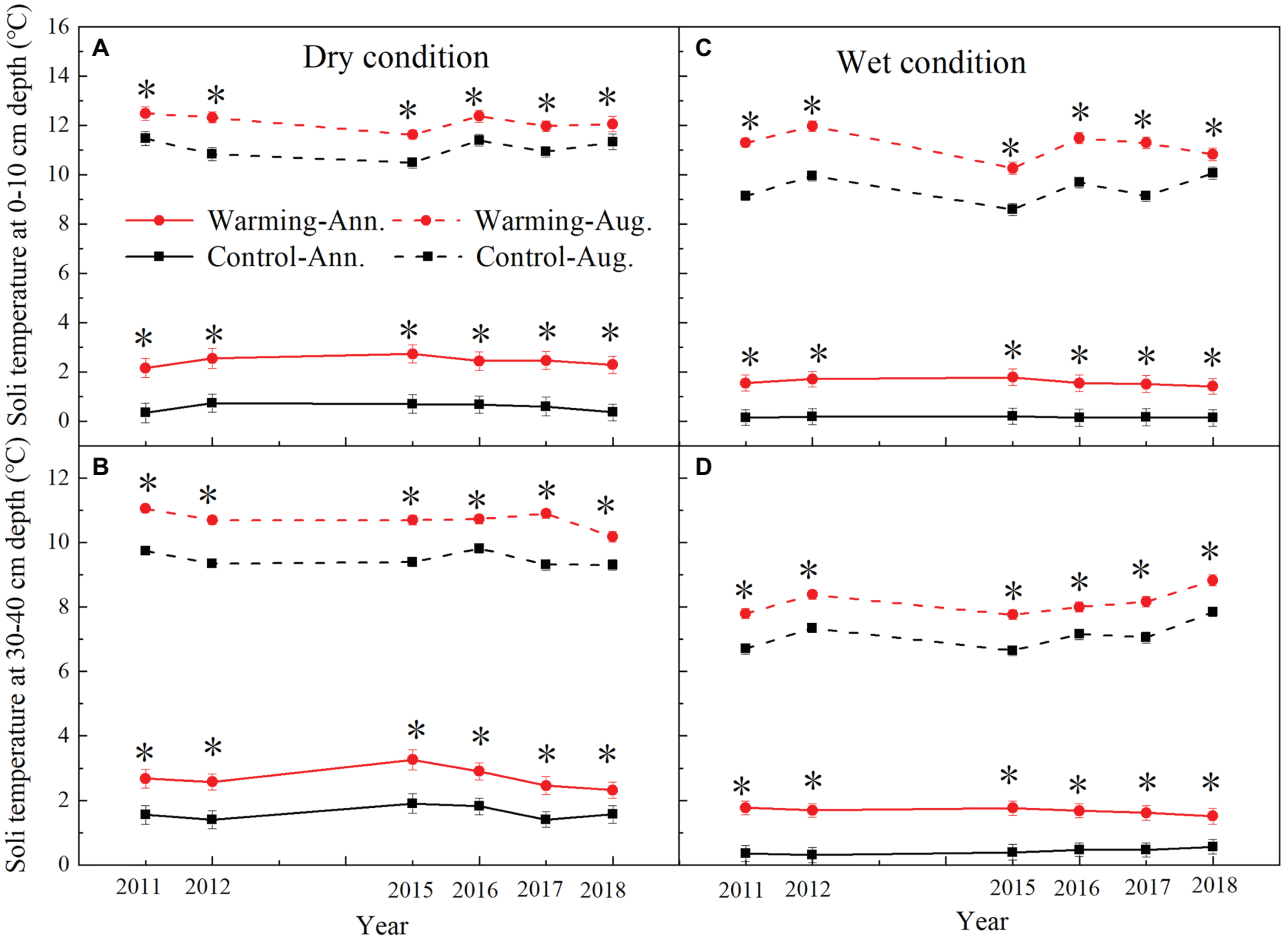
Mean August and annual soil temperature at 0–10cm and 30–40cm depth in control and warmed plots in the dry **(A,B)** and wet **(C,D)** conditions from 2010 to 2018. Significance: ^*^p<0.05.

The mean annual soil temperature was higher in the dry condition (0–10cm, 0.57°C; 30–40cm, 1.64°C) than wet condition (0–10cm, 0.16°C; 30–40cm, 0.40°C; Supplementary Figure S1; Figure 3) in the control plots over the study period. Warming has significant effects on soil temperature, which varied with soil depth and the soil moisture condition (Table 1). Warming treatments significantly increased the mean annual soil temperature. The largest increase was in 2015, which was 2.03 and 1.57°C in 0–10cm layer in the dry and wet conditions (Figure 3), respectively. The lowest increase was in 2017, which was 1.77 and 1.28°C in 0–10cm layer in the dry and wet conditions (Figure 3), respectively.

**Table 1:** Results (*F* values) of repeated-measures ANOVA of the effect of soil moisture condition (P), warming treatment (W), year, and their interactions on community aboveground biomass (AGB), AGB of grasses (Grasses), AGB of sedges (Sedges), and AGB of forbs (Forbs), and species richness (SR); the effect of soil moisture condition (P), warming treatment (W), year, depth, and their interactions on soil temperature (ST) and soil moisture (SM).

Variance source	ST	SM	AGB	Grasses	Sedges	Forbs	SR
P	**57.43** ^**^	**381.23** ^**^	**38.38** ^**^	1.67	**71.99** ^**^	**9.06** ^**^	1.21^**^
W	**146.49** ^**^	**4.35** ^*^	**2.41** ^*^	**21.22** ^**^	**3.37** ^*^	**10.79** ^*^	1.21
Year	1.91	**40.36** ^**^	**7.67** ^**^	**2.40** ^**^	**11.70** ^**^	**1.53** ^**^	**4.37** ^*^
Depth	**36.76** ^**^	**444.29** ^**^	/	/	/	/	/
P*W	1.23	**4.80** ^*^	**5.87** ^*^	0.10	**6.61** ^**^	**4.39** ^*^	0.02
P*Year	0.86	**10.19** ^**^	1.45	1.71	**1.91** ^*^	0.61	0.33
P*Depth	**11.94** ^**^	**53.31** ^**^	/	/	/	/	/
W*Year	0.50	0.21	**1.98** ^*^	0.34	**2.85** ^*^	0.85	1.12
W*Depth	**11.82** ^**^	**61.42** ^**^	/	/	/	/	/
Year*Depth	0.64	1.69	/	/	/	/	/
P*W*Year	0.06	0.65	0.12	0.24	0.15	0.17	0.41
P*W*Depth	**4.82** ^*^	**18.28** ^**^	/	/	/	/	/
P*Year*Depth	0.47	0.71	/	/	/	/	/
W*Depth*Year	0.19	0.49	/	/	/	/	/
P*W*Year*Depth	0.05	1.70	/	/	/	/	/

* *p*<0.05;

** *p*<0.01.

**Figure 4:**
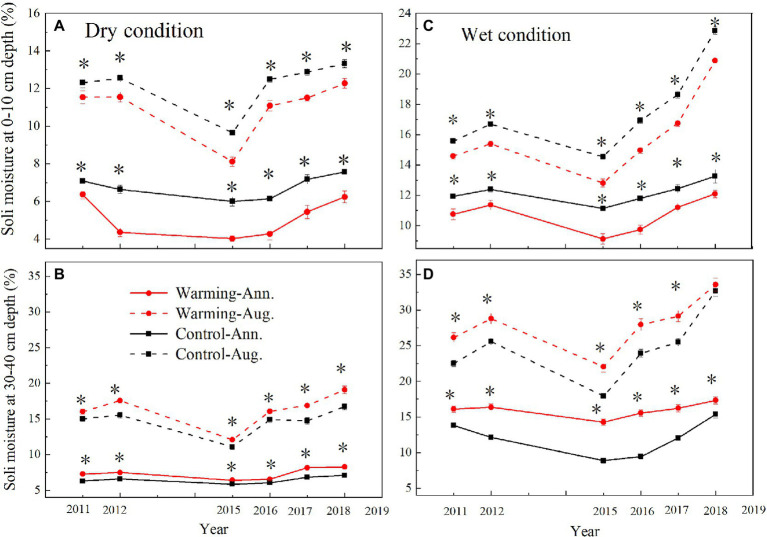
Mean August and annual soil moisture at 0–10cm and 30–40cm depth in control and warmed plots in the dry **(A,B)** and wet **(C,D)** conditions from 2010 to 2018. Significance: ^*^p<0.05.

The mean annual soil moisture was higher in the wet condition (0–10cm, 12%, v/v%; 30–40cm, 14%, v/v%) than dry condition (0–10cm, 6%, v/v%; 30–40cm, 8%, v/v%; Figure 4). Warming decreased the mean daily, August, and annual soil moisture in 0–10cm layer (Supplementary Figure S2; Figure 4), but increased it in 30–40cm layer (Figure 4). Warming significantly decreased the mean annual soil moisture by 0.71–1.28 and 0.95–1.92% (v/v%) in 0–10cm layer but increased it by 0.49–1.35 and 1.16–3.42% (v/v%) in 30–40cm layer in the dry and wet conditions (Figure 4), respectively.

### Aboveground Biomass

**Figure 5:**
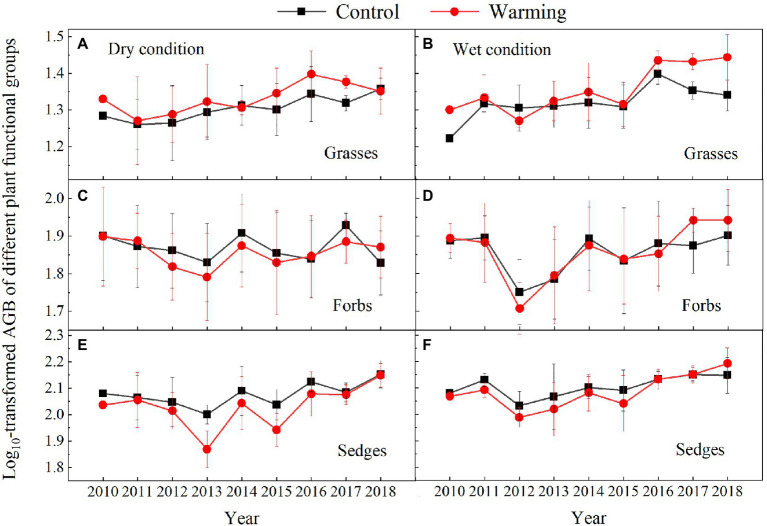
Aboveground biomass of grasses **(A,B)**, forbs **(C,D)**, and sedges **(E,F)** in control and warmed plots in the dry **(A,C,E)** and wet **(B,D,F)** conditions from 2010 to 2018.

**Figure 6:**
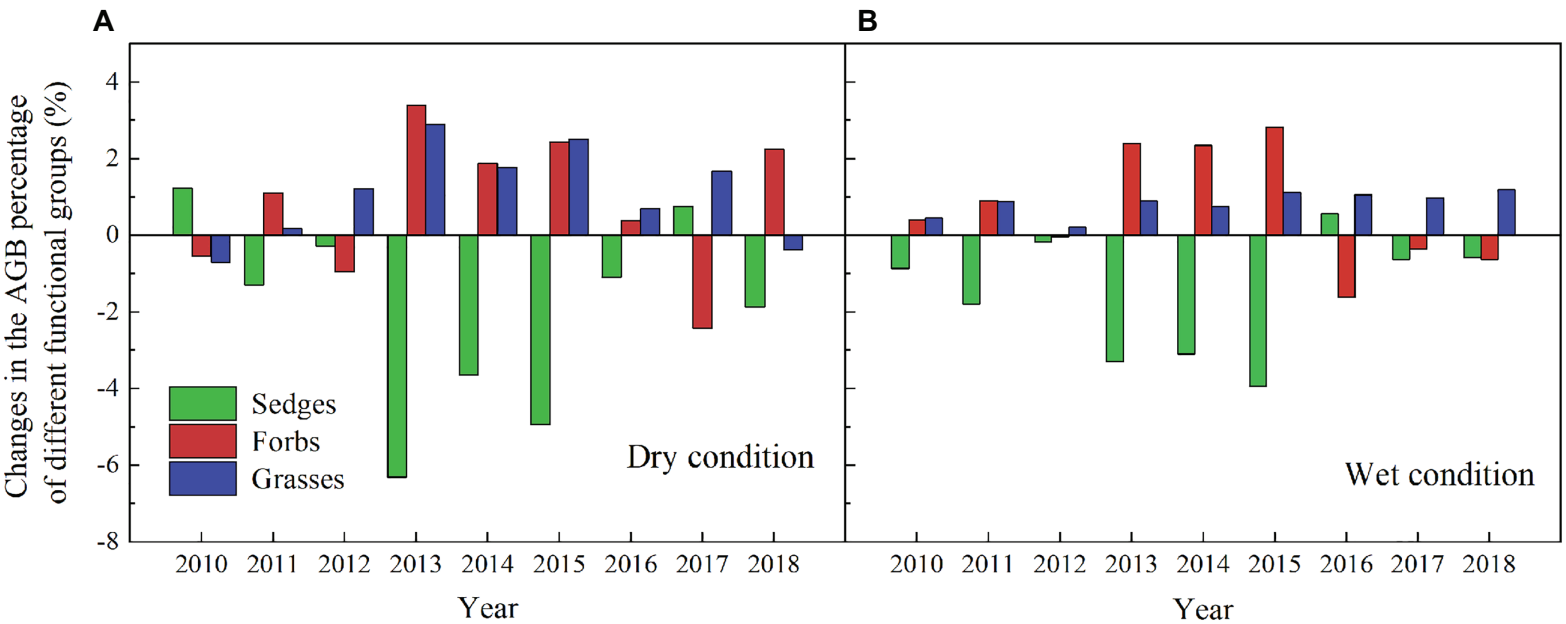
Changes in AGB absolute percentage of grasses, forbs, and sedges (percentage of different functional groups in warmed plots – percentage of different functional groups in control plots) in the dry **(A)** and wet **(B)** conditions from 2010 to 2018.

**Figure 7:**
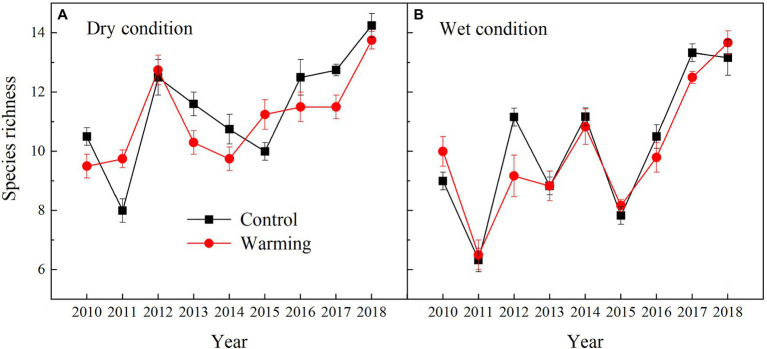
Yearly species richness in control and warmed plots in the dry **(A)** and wet **(B)** conditions from 2010 to 2018.

The AGB of grasses increased regardless of the soil water availability in the experimental plots, while the AGB of sedges decreased in the dry condition in the warmed plots during the experimental period (Figure 5). In general, the AGB percentage of grasses, sedges, and forbs were about 8, 55, and 37% in 2010 in the dry condition, and were 5, 63, and 32% in 2010 in the wet condition, respectively. The AGB percentage of grasses and forbs increased, while the AGB percentage of sedges decreased in the warmed plots (Figure 6). The largest increase in the AGB percentage of grasses and forbs was observed in 2013 and 2015 in the dry condition. The largest decrease in the AGB percentage of sedges was also observed in 2013 and 2015 in the dry and wet conditions (Figure 6). Warming had no significant effect on species richness in both dry and wet conditions during the experimental period (Table 1; Figure 7).

**Figure 8:**
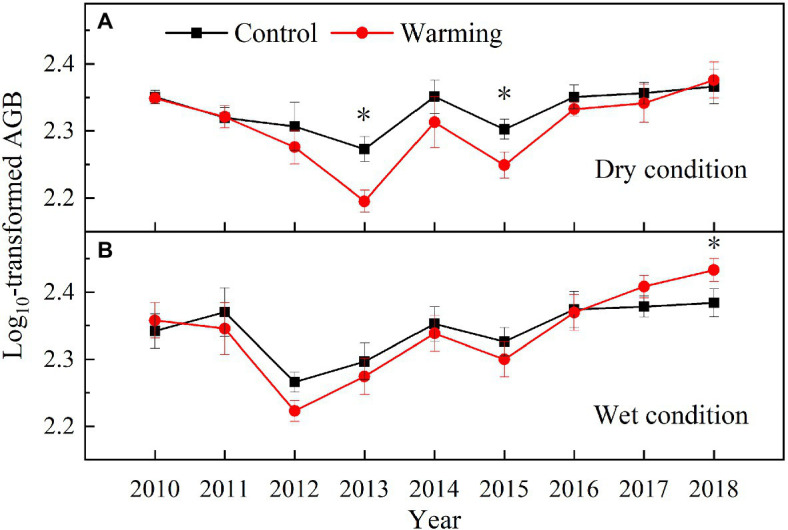
Aboveground biomass in control and warmed plots in the dry **(A)** and wet **(B)** conditions from 2010 to 2018. Significance: ^*^p<0.05.

Warming had a significant effect on the total AGB, which varied with the soil moisture condition and year (Table 1). The total AGB was higher in the wet condition than dry condition during the experimental period (Figure 8). It only significantly decreased by 16% in 2013 and 12% in 2015 in the dry condition (Figure 8A), but increased by 12% in 2018 in the wet condition, while showed no difference between warming and control plots in other years (Figure 8B).

### The Temporal Stability of Plant Community and Different Functional Groups Biomass

**Figure 9:**
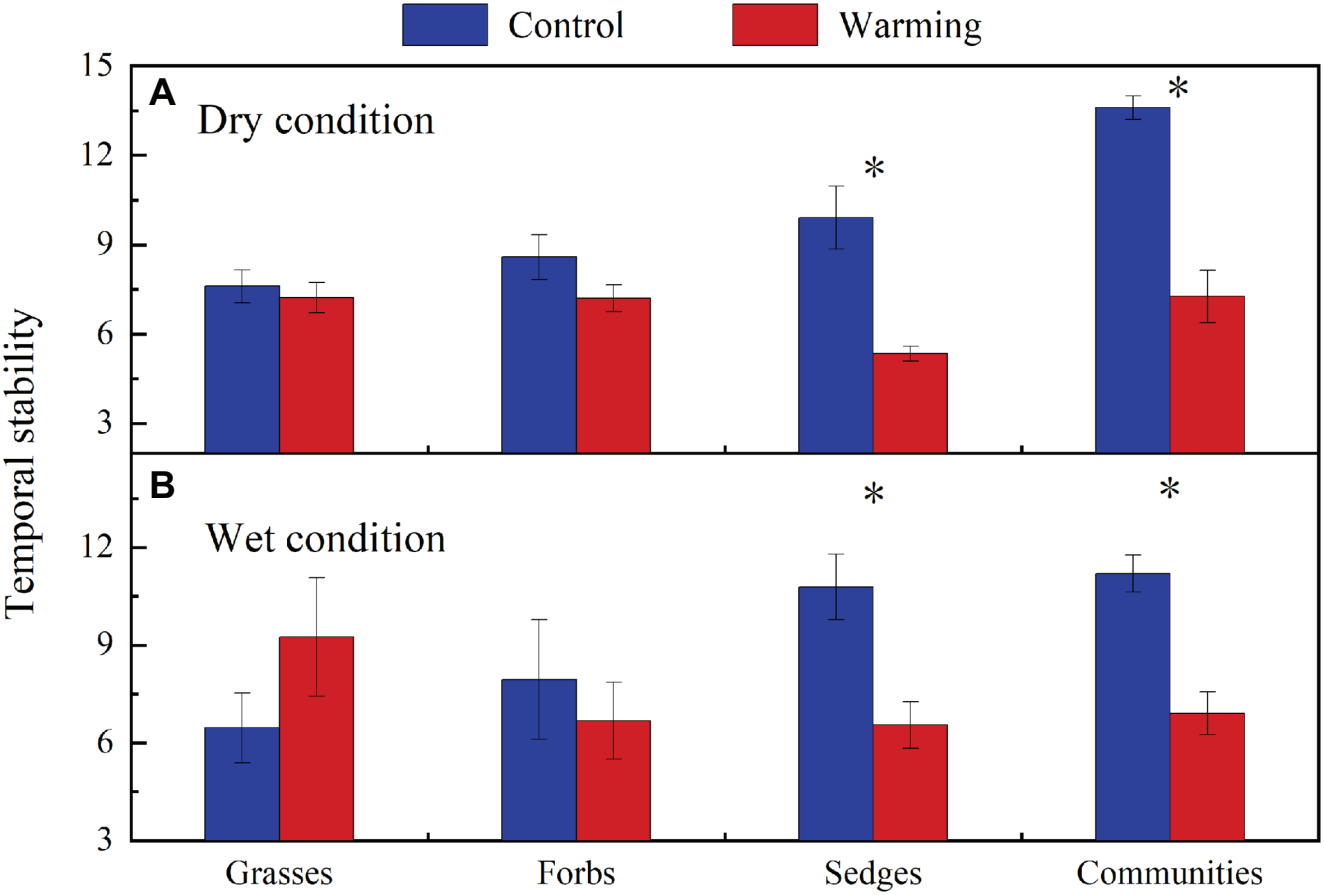
The temporal stability of plant community AGB and each functional group’s AGB in control and warmed plots in the dry **(A)** and wet **(B)** conditions. Significance: ^*^p<0.05.

Warming had a significant effect on the temporal stability of community AGB (*F*=28.51, *p*<0.001) and AGB of sedges (*F*=12.86, *p*=0.002). Warming had no significant effects on the temporal stability of AGB of forbs and grasses but significantly decreased the temporal stability of plant community AGB and AGB of sedges both in the dry and wet conditions (Figure 9).

### Relationship Between the Temporal Stability of Plant Community AGB and Species Richness and the Temporal Stability of AGB of Three Functional Groups

**Figure 10:**
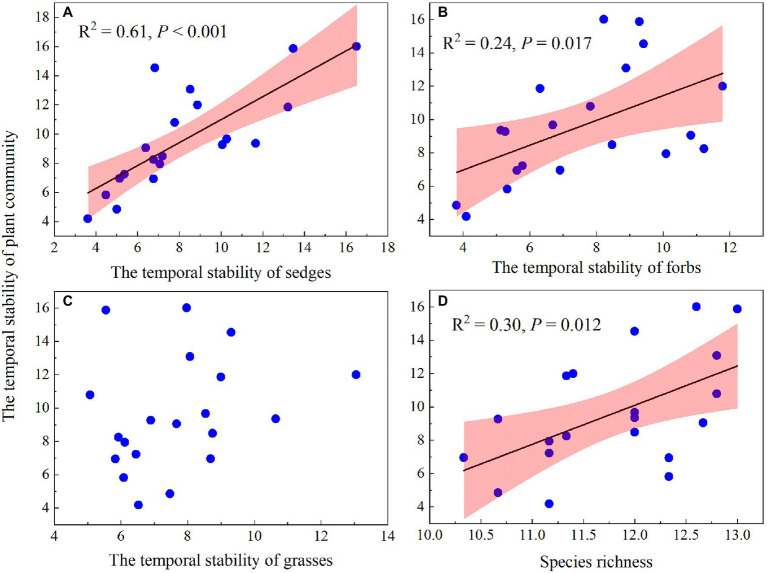
Relationship of the temporal stability of plant community AGB and the temporal stability of sedges AGB **(A)**, the temporal stability of forbs AGB **(B)**, the temporal stability of grasses AGB **(C)**, and species richness **(D)**.

The temporal stability of plant community AGB was positively correlated with species richness and the temporal stability of AGB of sedges and forbs (Figures 10A,B,D) but showed no relationship with the temporal stability of grasses AGB (Figure 10C). A GLM analysis showed that species richness and the temporal stability of AGB of three functional groups, together explained 79% of the variation in the temporal stability of plant community AGB (Table 2), with the largest proportion was contributed by the temporal stability of AGB of sedges (66.69%; Table 2).

**Table 2:** Integrative effects of species richness (RI), the temporal stability of AGB of sedges (Sedges), the temporal stability of AGB of forbs (forbs), the temporal stability of AGB of grasses (Grasses) on the temporal stability of plant community AGB in the dry and wet conditions based on the general linear models (GLMs).

Source	RI	Sedges	Forbs	Grasses
MS	7.23	134.13	15.73	1.20
SS%	3.60	66.69	7.82	0.60
*p*	0.183	**<0.001**	0.056	0.527

Bold values are statistically significant (*p*<0.05). MS, mean squares; SS, proportion of the total variance explained by the variable. *R*^2^=0.79.

## Discussion

### Plant Functional Groups Response to 9-Year Warming

Our 9-year warming experiment at an altitude of 4,630m in the permafrost region of the QTP found that grasses, forbs, and sedges show different responses to experimental warming (Figure 6). Similar to the long-term observations (32years) and a 4-year warming experiment on the eastern QTP (Liu et al., 2018), the AGB percentage of grasses and forbs significantly increased but that of sedges decreased regardless of the soil water availability in the experimental plots in our study (Figure 6). Different plant functional groups have different root traits, which may determine whether they have superior interspecific competition for limited resources in a warmer climate (Xu et al., 2018). Grasses and forbs species generally have roots deeper than 30cm, while sedges species have roots shallower than 20cm in our study site (Li et al., 2018, 2021). Warming induced a decrease in the soil moisture in the top layer (0–10cm) and an increase in the soil moisture in the deep layer (30–40cm; Figure 4). With the deep roots, grasses, and forbs species can access the soil moisture in the deep soil layer (Liu et al., 2018). By contrast, the deeper root systems and the increase in the soil moisture in the deep layer could make grasses and forbs species better able to cope with a warmer climate (Liu et al., 2018; Xu et al., 2020), thus leading to an increase in AGB of grasses and forbs in our study. However, sedges species growth would be inhibited because of the decrease in the soil moisture in the top layer caused by warming (Figures 4A,C; Liu et al., 2018). The largest decrease in AGB of sedges (27 and 20%) induced by warming in relatively drought years (2013 and 2015) suggested that the AGB percentage of sedges is more sensitive to precipitation than warming (Table 1).

However, our findings were inconsistent with the decrease of grasses abundance in an alpine steppe under a warmer climate (Ganjurjav et al., 2016), in which warming decreased the soil moisture only in the top layer and had no significant change in the deep layer. In our study, the increase of soil moisture in the deep layer (Figure 4) could create a favorable environment for the growth of grasses.

### The Community AGB Response to 9-Year Warming

The warming effects on community AGB varied with soil moisture condition (Table 1). Warming can affect plant growth directly and indirectly. The plant growth in alpine ecosystem is largely restricted by low temperature (Wang et al., 2012). The increase in temperature could directly alleviate the limitation of low temperature on plant growth (Li et al., 2018). An increase in temperature may indirectly stimulate plant growth by promoting soil nitrogen mineralization and increasing the growing season length (Sullivan and Welker, 2005; Peng et al., 2016). However, the associated soil moisture declining might suppress plant growth by aggravating water stress (Bai et al., 2004), which may offset the positive effect of increased temperature on plant biomass (Liu et al., 2018).

The decrease in the AGB of sedges species in the community can be partly compensated by the increase in the AGB of grasses and forbs species (Figure 6B). This might explain no significant change in community AGB during the normal and relatively wet years in the dry condition (Figure 8A). However, the largest decrease in soil moisture in the top layer constrained the growth of sedges (Figure 4A), leading to a dramatic decrease in AGB of sedges (27g·m^−2^) in relatively drought years (2013 and 2015) in the dry condition (Figure 5E). Simultaneously, the AGB of grasses only increased by 2g·m^−2^ (Figure 5A) in relatively drought years (2013 and 2015). Therefore, the decrease in community AGB was attributed to a rapid decrease in AGB of sedges under warming in relatively drought years in the dry condition (Figure 8A). Our results were similar with the finding reported by an alpine steppe on the QTP (Ganjurjav et al., 2016), while inconsistent with the results that suggest no significant warming effects on aboveground net primary production even during the drought years (Liu et al., 2018). In study of Liu et al. (2018), the soil moisture was only decreased by 11% under warming treatment, the decrease in soil moisture in the mesic alpine grassland might not limit the plant growth. However, warming decreased the soil moisture by 49% in the dry condition in relatively drought years (2013 and 2015; Figure 4A), which could constrain the plant growth.

Although, warming reduced the soil moisture in the top layer in the wet condition the decreased soil moisture might not limit the plant growth (Figures 5, 6B; Liu et al., 2018), which explains no significant warming effect on the AGB of sedges (Figure 8B). Grasses and forbs have deeper root systems and the increase in the soil moisture in the deep layer leads to an increase in AGB of grasses and forbs under warming (Figures 4D, 5). Therefore, the significant increase in community AGB (Figure 8B) was the result of the stimulation of grasses and forbs by warming in the wet condition in relatively wet years (Figures 6B,D).

### The Response of Temporal Stability of Plant Community AGB to Warming

Global climate change is expected to result in a high frequency of both drought and wet extremes, leading to changes in the temporal stability of plant community AGB (Zhang and Zhang, 2006; Xu et al., 2015). The decrease in the temporal stability of plant community AGB under warming in the dry condition in our study (Figure 9A) was consistent with previous findings from a 6-year warming experiment conducted in an alpine meadow on the eastern QTP (Quan et al., 2021), while inconsistent with the study reported by Zhou et al. (2019). Zhou et al. (2019) found there was no warming effect on the temporal stability of community AGB in an alpine meadow on the QTP. In study of Zhou et al. (2019), the decrease in the AGB of grasses species in the community can be compensated by the increase in the AGB of sedges and forbs species. The asynchrony among different plant functional groups makes the temporal stability of plant biomass remained stable under warming. However, the dramatic decrease in AGB of sedges (27g·m^−2^) in relatively drought years (2013 and 2015) in the dry condition (Figure 5E), while a minor increase in the AGB of grasses (2g·m^−2^; Figure 5), leads to a decrease of the community AGB (Figure 8A). Besides, warming had no effect on the community AGB in other years. Therefore, the temporal stability of plant community AGB decreased under warming in the dry condition in our study. The temporal stability of plant community AGB in the wet condition also decreased in the warmed plots (Figure 9B). Previous study suggests that warming increases the temporal stability of plant community AGB by increasing the biomass of dominant C_4_ functional group in the moist condition (Shi et al., 2016), primarily because C_4_ species have a higher tolerance to the water stress caused by warming (Way et al., 2014). However, in our study, the community is dominated by C_3_ plants that are more sensitive to warming compared to C_4_ species (Shi et al., 2016). Thus, the dominance by C_3_ plants may result in the decrease of temporal stability of plant community AGB in the alpine meadow ecosystem. The positive relationship between soil moisture and AGB of sedges has been widely reported by previous studies in the alpine ecosystem (Dorji et al., 2014; Liu et al., 2018). The interannual variability of soil moisture in the top layer was increased under warming (Figures 4A,C). As sedges species have shallow root systems (Liu et al., 2018), the interannual variability of AGB of sedges (Figure 8E) was also increased, which might explain that warming decreases the temporal stability of AGB of sedges.

The mass-ratio hypothesis suggests that the temporal stability of plant community AGB is mainly controlled by dominant species (Grime, 1998; Ma et al., 2017). Warming leading to changes in the temporal stability of dominant species AGB may greatly affect the temporal stability of plant community AGB. In our study, the AGB of sedges accounted for 55 and 63% of the community AGB in the dry and wet conditions in the control plots, indicating that community AGB may be determined by the changes in AGB of sedges (dominant plant functional group). The temporal stability of AGB of sedges accounted for the largest proportion (66.69%) of the variance for the temporal stability of plant community AGB (Table 2), and the temporal stability of AGB of sedges was positively correlated with the temporal stability of plant community AGB (Figure 10A), suggesting that the decrease in the temporal stability of plant community AGB was mainly resulted from the decrease in the temporal stability of AGB of sedges under warming.

Many previous studies, such as field observations (Tilman and Downing, 1994; Tilman, 1996) and theoretical models (Mougi and Kondoh, 2012; Loreau and de Mazancourt, 2013), have suggested that the temporal stability of plant community AGB increased with species diversity. In this study, we also found the temporal stability of plant community AGB increased with increasing species richness (Figure 10D). However, species richness was not a significant pathway through which warming can impact the temporal stability of plant community AGB (Table 2; Xu et al., 2015; Wu et al., 2020). The possible explanation for no effect of species diversity on the temporal stability of plant community AGB in our study is that the positive relationships of species diversity-stability were mainly found in experiments that have relatively large species diversity gradients (Tilman et al., 2006, 2014). In our study with a relatively small species diversity, warming had no significant effect on species richness regardless of the soil water availability in the experimental plots (Figure 7). Therefore, the warming effects on the temporal stability of plant community AGB through species richness in our study may be small.

In this study, we attempted to explore the effects of warming on the temporal stability of plant community AGB in the dry and wet conditions. However, there were only one dry stand and one wet stand. Thus, the different warming impact of temporal stability of AGB in the dry and wet conditions should be interpreted with caution, which needs to be further studied.

## Conclusion

Our results demonstrated that the community AGB had no significant change under warming in the normal years, it only decreased in the dry condition in relatively drought years and increased in the wet condition in relatively wet years. This suggests that the AGB response to warming depends on soil water availability. The temporal stability of plant community AGB is not driven by species diversity, but it is primarily regulated by the dominant plant functional group under warming. Our results suggest that the changes in the temporal stability of sedges species will directly affect the stability of natural grassland functions and services on the QTP under a warmer climate.

## Data Availability Statement

The raw data supporting the conclusions of this article will be made available by the authors, without undue reservation.

## Author Contributions

CLi, CLa, and FP had the main responsibility for data curation and writing. XX and FP had the overall responsibility of experimental design. QY, FL, PG, JL, and TW contributed to data analysis and reviewed this manuscript. All authors contributed to the article and approved the submitted version.

## Conflict of Interest

The authors declare that the research was conducted in the absence of any commercial or financial relationships that could be construed as a potential conflict of interest.

## Publisher’s Note

All claims expressed in this article are solely those of the authors and do not necessarily represent those of their affiliated organizations, or those of the publisher, the editors and the reviewers. Any product that may be evaluated in this article, or claim that may be made by its manufacturer, is not guaranteed or endorsed by the publisher.
